# Medical Care—More or Less?

**Published:** 1978

**Authors:** H. G. Mather

**Affiliations:** Consultant Physician, Southmead Hospital


### Medical Care ~ more or less?

**H. G. Mather, M.A., M.D., F.R.C.P.**
Consultant Physician, Southmead Hospital

### HISTORY

Edward Long Fox, B.A., D.M., F.R.C.P. was born in 1832 in Brislington. His father was a doctor, and both his uncle Henry Hawes Fox, and grandfather, Edward Long Fox, had been physicians to the Bristol Royal Infirmary. His grandfather founded an asylum in Brislington and was interested in mesmerism, but also achieved fame by having 22 children! With this background you will not be entirely surprised that Dr. Edward was elected to the staff at the age of 25, the year in which he qualified from Oxford! Consultants a century later are seldom appointed before the age of 35, but do not have to retire until 65, whereas Dr. Fox had to retire after 20 years on the staff, according to the rules of the day. He lived in Church House, Clifton and became medical officer to the College. He gave the Bradshaw lecture at The Royal College of Physicians in 1882 on 'The influence of the sympathetic on disease.' He wrote articles on chorea and the spleen, central nervous system diseases and phthisis.

He was an able, laborious, unselfish and generous Physician and philanthropist — 'He loved his profession and fellow creatures'. He devoted himself to stemming an outbreak of typhus in East Bristol, to the detriment of his small but growing practice. He was interested in tuberculosis and helped promote the establishment of Winsley Sanatorium. He realised the importance of a medical library and raised £1,200 at a dinner in Queen's Hotel, Clifton in 1888. He was a member of the Bristol Medical Reading Society which had started in 1807 and still thrives today. Not least of his generous actions was the annual strawberry tea party which he gave to medical students and practitioners.

'He had the knack of appearing to consult his senior pupils in the way which was very flattering, and even when he did not accept their opinions, he was so adroit that students frequently thought he was following their suggestions when, in fact, he was adopting quite a different line of treatment' — he should have been a politician!

He suffered from gout for 20 years — alcohol could not be blamed as he was abstemious, and President of the National Temperance League! He gave us an insight into the psychosomatic factor in disease when he wrote 'There is nothing to bring on a fit of the gout like a run of anxious cases'. In the last few years of his life he developed diabetes, then polyneuritis and leg vein thromboses and finally died with a dilated heart and a mitral bruit at the age of 70.

The first Long Fox Memorial Lecture was given in 1904 by Dr. John Beddoe who took as his subject 'The Ideal Physician'. Since then, the subjects have been extremely varied, including dentistry, surgical and medical advances, history and even the zoo. I have chosen to discuss with you whether medical care should demand more or less of our resources.

### COSTS OF MEDICAL CARE

The costs of running the Bristol Royal Infirmary a century ago when Long Fox was on the staff were £15,580/4/7 for one year. (State of the Bristol Royal Infirmary 1870). This catered for 216 beds with an average stay of 29 days and 18,816 out-patients. In 1877 a House Physician was appointed at a salary of £100, thereby increasing the junior staff to 3. The income was derived from charity and from subscribers. For two guineas a year the subscriber was entitled to recommend two in- and six out-patients per year. It was some time before medical officers took priority over subscribers' notes in deciding admissions!

Some of the rules were interesting:

1. Patients for admission to attend at 11.00 a.m., clean, and to bring a change of linen.
2. A patient still in the ward after 2 months to be discharged unless consultation of physicians and surgeons deem probability of further benefit.
3. If the patient lives more than 10 miles away, a deposit of £1 shall be made to help pay transport home or funeral.

Some of the expenses of running the Infirmary were interesting: the cost of meat was £400 more than all the wages of nurses, cleaners, porters, dispensers together. The total salaries, including doctors, secretary, matron, steward and dispenser were practically the same as the expenditure on beer, wines and spirits (£744).

The current cost of the National Health Service is £6,000 million per year. One sometimes hears the clamour for more money to be spent on health. If one takes the USA as a comparison, there is very little evidence that the vastly increased expenditure in that over-medicalised country improves health, happiness or life expectancy (Table 1).

**Table 1**

| | *US* | *UK* |
|---|---|---|
| Health Bill | $83 billion | £6 billion |
| Specialists | 280,000 | 8,000 |
| General Practitioners | 70,000 | 22,000 |
| Population Proportion | 4 | 1 |

The spiralling cost of health services has affected all countries. As an example, in the USA, the price index rose by 74% in 20 years, but the cost of medical care by 330%; health administrative costs increased sevenfold in 8 years and laboratory costs fivefold. In that country the burden falls on the individual via his insurance company. The cost may surprise you: a friend recently sent me details of a three day admission in Nevada for intestinal 'flu which cost $925,10.

In this country we are cushioned from knowing the costs, but it might be a worthwhile exercise if both doctor and patient knew for instance that ambulances cost £6 per mile, out-patient attendances cost £10, a day in hospital £40, a week's course of gentamycin £60, and a coronary arteriogram £400. In West Germany the cost of health care is £450 per head per year — four times our own costs. Over-investigation and over-treatment are rife; Kurs in fashionable resorts are prescribable under the national insurance. Because of the item of service system of payment, the average GP earns over £50,000 after deduction of all expenses. Even in that prosperous country the burden of health costs have provoked government attempts to prune it, by curtailing the excessively long hospital stay, over-prescribing and over-treatment. This has been met by sporadic strikes by medical and dental practitioners, who are among the highest paid of all professions.

One mentions these facts about medicine in Germany and the US to remind ourselves that we need in this country to put our own house in order as our costs are escalating in a similar way. In my opinion the government, which in a democratic society represents the will of the people, has a right to limit the amount of money devoted to medical care. There should be no restriction on what an individual pays out of his own pocket, but there is so much waste when the costs fall on the government.

**WASTE OF RESOURCES**

There has been a tremendous increase in the number of administrative staff following reorganisation of the NHS (16,400 according to the Public Accounts Committee) at an increased cost of over £52 million annually. From Hansard I take these figures: there were 700 non-medical employers at regional and district level earning £5,000 before reorganisation, and 4,800 after. The comparable figures for £8,000 salaries were 60 and 1,700. There is no evidence that I can find of improved efficiency to match this.

There is abundant evidence of incompetent administration; work on the new Liverpool Teaching Hospital started in 1968 and the hospital was to be opened in 1974 for a cost of £11.8 million. There is no immediate prospect for completion and the estimated cost is now £54 million.

The Duke of Edinburgh speaking in Cambridge the other day said 'Some people believe that industries really exist for the benefit of those employed in them first, and their value to the consumer second.' and this may be pertinent to the expanded bureaucracy running the 800,000 people employed in the Health Service, but he also said 'Britons suffered from people finding fault with others without seeking to improve their own performances.' This brings me to the nub of my lecture — whether the medical profession itself cannot do a lot to contain costs rather than just sniping at the civil servants.

**JUNIOR DOCTORS**

There has been an increase in hospital medical staff ten times greater than the increase of population. If this rate were sustained, by the year 2183 everyone in the realm from cradle to grave would be a hospital doctor! Despite larger numbers there has been this tremendous cost of overtime for junior staff — £1 million a year in Avon alone. Unfortunately, the system has led to abuses such as excessive numbers of juniors being on call instead of sharing duties, and the claiming of A Units of Medical Time by staff who are hardly ever involved in emergency work. I even heard of one example where 2 Units a week were being claimed for organising clinical meetings! This system has also led to the anachronism whereby a senior registrar may have to drop his salary by £2,000 on being promoted to the more onerous task of a consultant.

TRAINING

Concerning the training of doctors, Paul Beeson in 1974 found that there were 69 full-time and 114 part-time teachers in Oxford, compared with 840 and 1129 respectively in Harvard. He wrote 'I had previously allowed myself to be persuaded that didactic lectures are a waste of time and that, the more teachers one can have in the hospital, the better the learning opportunity of the students. The surprising thing is that I do not find much difference in the products of the two systems'. I consider that in this country the government is right, in its Resource Allocations Working Party, to attempt some devolution of medical resources from the big centres. It is, of course, essential to preserve centres of excellence for research and development.

Medical schools throughout the world see themselves as centres of excellence, with high technology and high staffing ratios, but I sometimes wonder whether in consequence they are the best places for undergraduate education. One tends to agree with the Director General of the World Health Organisation, Dr. H. Mahler, when he says that over-emphasis on the most expensive technologies leads to education in medical schools becoming insensitive to the health needs and problems of the community.

In developing countries there is a real danger that medical students are taught high technology Western medical methods instead of the more practical measures appropriate to the problems in that country. The large teaching hospital is sometimes merely a status symbol, as sometimes much of the apparatus is unused through lack of trained staff or proper servicing. The graduates of some of these medical schools gravitate to the cities and compete harshly for patients, while the rest of the country needs drains not drugs, extra food not X-rays, houses not monitors.

**DRUG COSTS**

We are constantly being reminded in this country of the cost of the pharmaceutical side of the NHS, and there is undoubted over-prescribing, prescribing of more costly preparations with no benefit over the pharmacopoeal equivalents, and waste of expensive drugs in bathroom cupboards. In the USA, where most people actually pay for their medicines, it is difficult to justify the consumption of 26,000 tons of antibiotics annually at a cost of $2 billion. In one survey half the practitioners prescribed antibiotics for the common cold — perhaps the fear of litigation had some influence in this. Every 24 hours from 50—80% of adults in the US and UK swallow a medically prescribed chemical — sometimes the wrong one, a contaminated or old batch, a counterfeit, or in dangerous or ineffective combinations. You will not be surprised to hear that the cost of the pharmaceutical services is one and a half times that of the family doctors. Dependence on prescribed tranquillisers has risen 290% in 10 years, whereas consumption of liquor only rose 23% and opiates 50%. I am told that in the US one should diagnose diazepam deficiency!

Could it be that this medically mediated dependence is in part the result of promotion by drug firms? You can visit Brussels on a beta-blocker, lunch on lorazepam and dine on diazepam! One seriously wonders whether an advertisement in The Lancet of 1st October proclaiming that Trasylol (aprotinin) 'increases the chances of survival in acute pancreatitis' contravenes the Trades Description Act when, in a previous number, a controlled trial had shown no such benefit. It is perhaps sobering to realise that the US pharmaceutical industry spent an average $4,500 per doctor on advertising and promotion in 1972. It would do us all good to be marooned and to be allowed to choose only 10 'desert island drugs' rather than being allowed to choose from the 3,120 listed in MIMS and the 580 in the National Formulary. In West Germany there are 2,400 pharmaceutical firms producing 30,000 different proprietary drugs so that I become very sorry for the practitioner and even sorrier for the patient.

### DRUGS

The medical profession has become much more aware of the ill-effects of drugs since the disaster with thalidomide. However, we need constant reminders, for example, of the 44 fatal aplastic anaemias produced by phenylbutazone and oxphenbutazone in one year (Inman 1977), and of the serious problem of analgesic nephropathy. We need to read publications that spell out the hazards of hormone replacement therapy for the menopause, where the risk of endometrial cancer is increased between four and eight times compared with controls. Oestrogen therapy for prostatic cancer relieves pain from bone secondaries, but does not prolong life, due to the adverse thrombotic effects of the drug.

Of equal importance to the potentially serious complications of drug therapy are the prescribing of remedies without proven value. An example is ergotamine which is incorporated in innumerable preparations for the treatment of migraine. Where controlled observations have been made, as by Waters (1970), 40 benefited from ergotamine, 46 from placebo, and ergotamine aggravated the attack more frequently than placebo.

From what I have said already, I hope you will agree that we could achieve considerable savings by more economical prescribing. Not the least of the benefits should be a reduction in the 1% of hospital admissions now attributed to drug reactions, and the 8% incidence of adverse effects.

**RANDOMISED CONTROLLED TRIALS**

These become increasingly more important with the advent of powerful and sometimes harmful drugs, the increasing cost of medical care in general and the natural desire of doctors to use the best possible remedies for their patients. Any student of medical treatment will be aware of the fads and fashions which have dominated the management of, for example peptic ulcer. Innumerable dietary regimes, antacids, antispasmodics and operations have been advised by authoritarian doctors over the last century. Only recently have controlled trials shown, for instance that dietary alterations for duodenal ulcer carry no benefit in healing, and that the safer operation of vagotomy and pyloroplasty is better than gastrectomy. There are however still far too few proper trials of therapy; for example between 1964 and 1974, of 35,228 publications in the Index Medicus on therapy in gastro-enterology, only 0.9% were randomised controlled trials (Juhl et al 1977). We can take some credit from the fact that the largest number originated in the United Kingdom.

When a specific remedy is found for disease, such as insulin or vitamin B12, trials are only necessary to determine the most effective dose and mode of delivery. But in rare diseases such as leukaemia, proper studies are essential, on a multi-centre basis, in order to improve our management. The drugs used are very expensive and toxic, there is a high consumption of blood products, nursing and medical time, and the results of treatment are not clear cut. Randomised trials have resulted in a better prognosis for acute lymphoblastic leukaemia in children, but there have so far been less spectacular improvements in adults with acute myeloid leukaemia. In the anaemias resulting from bone marrow failure, randomised controlled trials have shown that nandrolone confers no benefit compared with placebo (Branda et al 1977). Such studies are helpful not only because they save the patient unnecessary drugs with potential side effects, but teach us how dramatically some will recover spontaneously.

In the field of cardiology many treatments have been introduced in the last thirty years which have since been found to confer no significant benefit. Anticoagulants (Tulloch & Gilchrist, 1950) were reputed to halve the mortality from acute myocardial infarction until an MRC working party report (1969) showed their lack of value. The rat poison (warfarin) might perhaps have been better used to conserve food stores in the third world! The use of glucose, potassium and insulin was reported by Mittra in 1965 to halve the mortality in coronaries until it was refuted by randomised controlled trials in 1968 (Pentecost et al). The routine use of lignocaine has been advocated in the management of acute heart attacks, and the manufacturers have not been slow to encourage this. Controlled trials have shown that there has been no alteration in mortality (Darby et al 1972; Mogensen 1970), though the suppression of ventricular extrasystoles has no doubt tranquilised the attendant doctor and nurse.

These three examples of active treatment for the acute heart attack have persuaded the medical profession that hospital and coronary care units are necessary for their management. A joint general practitioner/hospital study in the South West of England showed however (Mather et al 1976) that where random allocation of patients was possible, home-treated patients fared as well. Moreover, those over 60 years of age had a better prognosis in home circumstances. These observations are now being repeated in Nottingham with similar though as yet unpublished results.

One final example in the field of cardiology is the study of the duration of bed rest needed in treating heart attacks. John Beddoe in the first Long Fox memorial lecture in 1904 said 'An exaggerated respect for authority is an impediment to the progress of medicine'. We were taught that the victim of a coronary had to rest for six weeks in bed, being fed and washed for much of the time. Properly controlled randomised trials, most originating in this country, have shown that patients fare just as well after but a few days rest (Hayes 1974). The mind boggles at the cost of the unnecessary enforced rest, with complications such as embolism, pneumonia, urinary retention, bed sores, osteoporosis and cardiac neurosis which this medical authoritarianism caused.

I think it is not unreasonable to conclude from the examples quoted that randomised controlled trials are not only ethically justifiable, but can show a great saving in drug costs and hospital stay. Perhaps they should be obligatory before any new treatment is accepted, and should be applied to many that are taken for granted. As an example, in the only randomised trial that I know for physiotherapy, there was no difference in the improvement of painful arthritis compared with placebo (Hamilton et al 1959).

### ADVANCES IN MEDICINE

In the last century, the most striking advances have been in the control of communicable diseases (McKeown 1976) Smallpox has been practically eradicated from the world, thanks to the pioneer work of Edward Jenner of Berkeley. The epidemics of plague, typhus, cholera, and enteric fever have been eliminated from the Western world, not by vaccines or antibiotics, but by the sanitary and water engineers, the builders of houses and food stores. Doctors played their part in discovering the causes of these diseases and their mode of spread, an example being William Budd of this city. I think we pay insufficient tribute to the inventors of the water closet and sewage works.

The decline of scarlet fever and diphtheria owe more to improved housing, less overcrowding and better nutrition than to medical treatment or immunisation. Professor Bruce Perry, in a Long Fox Lecture (1944) pointed out how rheumatic fever was related to social circumstances; abolition of overcrowding has done as much to control this scourge as have the antibiotics. Vaccination against poliomyelitis can however be counted as a success for medical intervention in what used to be an untreatable disease.

The decline in incidence of tuberculosis has been steady in the last century, thanks to safe milk, better nutrition and less overcrowding. The advent of chemotherapy hastened this decline in the last 30 years and is one of the few examples of really successful therapeutics affecting mortality trends. One must pay tribute to our chest physicians, whose controlled trials have been a lighthouse to the rest of the world. In achieving this remarkable control of most communicable diseases, the doctor's role has largely been in research (by bacteriologists, epidemiologists and chemists) and in the educational and advisory role of medical officers of health and others to the state. The diseases of poverty such as malnutrition, rickets and scurvy have been eliminated by better pay, food and education rather than pills and potions.

The control in the last century of so many communicable diseases has been an extraordinary epoch in world history, but, as Sir George Godber said recently — 'We've done the easy part' and he sees no miracles on the medical horizon. 'The next stage was persuading individuals to change their ways of life.' Only by such persuasion can accidents, and diseases caused by alcohol, tobacco, drugs, gluttony and lack of exercise be brought under control. These maladies consume a considerable proportion of our health resources. Much of the rest is used in coping with hereditary or congenital disorders, neoplasms and degenerative conditions for which, in general, only palliation is possible.

Dr. Fox was well aware of the educational function of the doctor, and in fact his Presidential Address to the British Medical Association when it met in Bristol in 1894 was 'Medical Man and the State' (Fox, 1894). He felt that the doctor should advise the state for the betterment of the individual, especially the poor, the sick and the criminal. He should educate on sanitary laws, water supply, healthy houses, noxious trades and the ill effects of gluttony and drunkeness.

Doctors are now in a different position, since the state is a near monopoly employer and there is no financial restriction for the patient seeking advice or treatment and the doctor giving it. We have to advise whether the *State* should spend more on medical care which means more medical and para-medical professionals, bearing in mind the demands of schools, police, road safety, food supply etc. Then within this limited budget, we have to assess relative priorities and cost effectiveness. It is therefore important to examine some of the technological advances in medicine critically.

### ENDOSCOPY

The development of instruments and techniques since the war has been outstanding, and I would here like to pay tribute to the medical and surgical staffs of the Bristol hospitals for their skills. There is no doubt of the value of endoscopy as a research procedure. What is not yet certain is the role of endoscopy in the management of patients throughout the country, as it is costly in apparatus and medical time. Kern (1976) in a critical review questions whether the visualisation of a mucosal lesion decreases morbidity or mortality.

Colonoscopy has been advocated for 'unexplained colonic symptoms', 'chronic abdominal pain' amongst other indications, but how often should the procedure be done and by whom? He goes on to say 'society pays more for a procedure or operation than for a careful critical history, physical examination and a thoughtful discriminating formulation of diagnosis and management, even though the latter requires far more training and experience and much more extensive knowledge and understanding'. In a randomised trial in Nottingham, Dronfield et al (1977) found no difference in management or survival in acute upper gastro-intestinal bleeding whether investigation was by gastroscopy or radiology, and the accuracy of findings at surgery or necroscopy was similar. Thus there is no present indication for all patients to be subjected to gastroscopy if good radiological facilities are available. The remarks of Halberstam (1976) from Washington may be pertinent. 'Mallory climbed Everest because it was there but I'll be dammed if all my patients' orifices have to be cannulated because they are there! Too many elegant diagnostic techniques are great feats of dexterity and technology, but can at best give us only partially complete information. We can no longer afford them'.

**COMPUTERS**

All of us have experience of apparatus bought in a wave of enthusiasm which gathers dust from disuse, either in the laboratory, theatre, clinical side room or special department. With the increased cost of medical hardware, the potential waste of money is that much greater. A computerised record system in King's College Hospital was tried and failed many years ago. Now I read of an expensive computer in Exeter which cost nearly a million pounds and whose running expenses for salaries and maintenance are half a million per year. It is most important that we are told in a few years time if there have been any benefits to the citizens of Exeter in improved health, or a comparable reduction in the humber of record clerks to match this sum! For the same annual sum I estimate that 961 elderly subjects could have home help for two half days a week or one could employ 77 staff nurses and 90 district nurses.

**ELECTROENCEPHALOGRAPHY AND EMI SCAN**

The EEG has been and remains a useful tool in neuro-psychiatric research. In the routine clinical field it may be of value in confirming petit mal and perhaps in determining whether repeated 'attacks' are functional or organic. It's value in the diagnosis and management of grand mal is unproven. Hopkins & Scambler (1977) in a review of epilepsy from St. Bartholomew's Hospital found that half of the subjects had a normal EEG and that 5 of 12 patients with repeated fits never had an abnormal EEG. The tracings did not in any of their 94 patients aid management and, in particular, did not point to the need for further investigation in 4 who had cerebral tumours.

By contrast, the EMI Brain scanner, though an expensive item to instal, has been shown to be not only accurate in diagnosis, but cost-effective (Thomson 1977). Balancing the cost of the machine and extra staff have been the reduction in the number of invasive investigations such as arteriograms and air studies, a reduction in waiting lists with an increased turnover of patients. By offsetting the cost of invasive techniques which were not done, and the beds which were not used, Thomson showed that there had been a considerable nett saving to the National Health Service of perhaps £36,000 in 1975/76.

**HEALTH SCREENING**

This new concept in health care is based on the premise that it is worthwhile discovering an abnormality which was not producing symptoms sufficient to take that person to a doctor. Therefore, it presupposes that the abnormality is correctable with benefit to that person's health, otherwise we are just making people patients without being sick (Illich 1975), and breeding demands for medical services. Cervical cytology has been shown to be marginally effective in detecting cancer of the cervix in its pre-invasive phase. The prevalence of the disease was already waning before mass screening was introduced; the problem is in getting those at greatest risk to be examined. There is no evidence that the early discovery of glycosuria alters the natural history of diabetes, or the finding of an abnormal ECG or cholesterol alters the management of ischaemic heart disease. There is a real danger of producing a new group of cardiac neurotics who count cholesterol to replace the unwarranted invalidism produced by a previous generation of doctors who restricted healthy people who had innocent murmurs or neuro-circulatory asthenia.

The situation in hypertension is probably rather different. There is growing evidence that treatment of asymptomatic hypertension confers significant benefit to the individual, particularly by reducing the frequency of strokes. One problem is that of patient compliance; most of the studies have been on selected groups such as the Veterans Administration Co-operative Study (1972) where non-co-operators were excluded. Another is to detect those likely to benefit, and here surely the existing organisation of general practices could cope, rather than developing separate screening units with their inevitable administrative costs and lack of job satisfaction. The frequency of blood pressure checks required varies with the age and previous recordings. The cost of increased life exectancy varies with age and inversely with diastolic pressure, so that it is relatively more expensive to give benefit to a 60 year old man with a diastolic level of 110mm.Hg. than a 40 year old man with 120mm. (Stason & Weinstein 1977).

### PATHOLOGICAL TESTS

Particularly since the availability of automated analysers in biochemistry and haematology, there has been an explosion in the number of tests done on patients without any evidence of benefit in their safe management (Brod 1977). Surely here is an example of health costs escalating because of indiscriminate use of a facility because it is available. A study from Rochester, N.Y. showed that frequently resident staff did not even check the results of the tests which they had ordered (Griner & Liftzin 1971). Apart from the abuse of some of these routine tests, we are in danger of placing excessive reliance on more sophisticated and expensive radio-immune assays. For example our ability to measure levels of digoxin in the blood does not mean that all patients being treated with digoxin need such tests, or that a parathormone estimation is necessary before removal of a parathyroid adenoma. I must make it clear that I am in no way opposed to the use of elaborate and even unnecessary techniques if they are undertaken as part of a planned research programme.

### THE ELDERLY

Life expectancy at 65 has hardly changed in a century — surely driving home the fact that medicine can do little for arthritis, most cancers, arteriosclerosis and ageing. However the proportion of our population over 65 is increasing steadily.

Resources for the elderly have been allocated in increasing amounts, but have they been applied in the right places? In Manor Park Hospital, Bristol there were 1798 admissions in 1956, and 1920 in 1975, but in that period medical staff has doubled, there have been 10 more social workers and seven times the number of occupational therapists. Although the number of beds has fallen from 733 to 525, the cost of running the hospital has increased tenfold. One wonders whether money for these staff could have been more profitably applied to providing home helps and home nurses.

Except for those concerned with maternity and child health, the care of the elderly forms an increasing part of all our practices. A lot can be done to improve the quality of life, for example by chiropody, hearing aids, control of diabetes and heart failure, and hip replacements. Quite rightly some consultants have specialised in the problems of the elderly, and have pointed out deficiencies in care, and forged good links with local authority services. Unfortunately in many parts of the country, enthusiasm has led to the development of an entirely separate organisation for those over 65, with reduplication of junior staff and wicked waste of resources. Surely we should work towards better integration and hence economy. We should no more expect a geriatrician to care for all over 65 than a chest physician to treat all bronchopneumonia or an orthopaedic surgeon all backache.

### CHILD HEALTH

The recent report on antenatal screening for maternal serum-alpha-fetoprotein in order to predict anencephaly and spina bifida (UK collaborative study on alpha fetoprotein, 1977) sounds a hopeful note that it may be possible to prevent these congenital malformations by timely abortion. If larger experience confirms this, then the procedure should prove cost-effective and we should no longer need to undertake the expensive and unsatisfactory operations to improve the quality of life of these unfortunate children.

Studies in the department of Community Medicine of St. Thomas's Hospital (Melia et al 1977) are a good example of potentially fruitful research in preventive medicine. These workers found that children in homes where there was cooking by gas had more bronchitis and wheezing than those living in homes with electric cookers. Similar studies in Hong Kong have shown that fumes from paraffin stoves may cause premature lung cancer in young people.

The story with regard to breast feeding is however not a credit to modern medicine. Western habits of bottle feeding have spread to the developing world, producing not only impaired child health but economic problems. For instance in 1960 in Chile 96% of mothers fed their babies for a year or more. A decade later only 20% breast fed for more than 2

months. It has been estimated that 32,000 cows are needed to produce the deficit!

**X-RAY INVESTIGATIONS**

There have been important advances in diagnostic radiology in the last few decades, and reference has already been made to the EMI scanner. It behoves surgeons and physicians to be self critical in the ordering of X-rays not only because of the radiation hazard involved but because of the cost of equipment, radiographer's time and films. The question should be asked 'Will this examination alter the management of the patient?', rather than requesting it as a routine, or because of clinical curiosity. Intravenous pyelography may be taken as an example: it used to be advocated as a routine in the investigation of hypertension. Critical evaluation has shown that the yield in terms of remediable disease is so small as to be worthless, and the test should be reserved for progressive hypertension in the young person who gives no family history of hypertension and in whom there are clinical features to suggest renal disease. The IVP is likewise done routinely by most surgeons before prostatectomy. Wilcox & Mitchell (1977) have however shown that the IVP, which had been performed in 82% of patients in acute retention, neither influences the decision to operate nor the type of operation. It was a disservice to patient and hospital in that the average delay before operation was 8 days, compared with 3.5 days in those who did not have an IVP.

You may think that I am being unduly critical but the problem is very real — demands increase in the UK at the rate of 5–10% per annum and many radiologists are emigrating. One quarter of pregnant women are x-rayed despite the advent of ultrasound, and in the USA two thirds of the whole population was x-rayed in 1970! I am glad to report that the Royal College of Radiologists is looking in to these problems of medical audit.

### SURGERY

Length of stay in hospital after operation has varied, as much from the habits of the ward sister or surgeon as to the needs of the patient. However, it has been shown by Doran et al (1972), Ruckley et al (1973) and Lord (1969) that day case or 48 hour admissions for hernias, varicose vein and haemorrhoid operations are not only feasible but have considerable social and financial benefits without hazard to the patient. Fegan (1963) revolutionised the treatment of varicose veins when he introduced compression sclerotherapy on ambulant patients, and recently McGarry (1977) has reported on 200 out-patient terminations of pregnancy, and Boardman & Griffiths (1977) on 588 out-patient orthopaedic operations in two years. The advantages are not only economic, but a greatly reduced waiting time, and satisfaction for the patient at not having to sleep away from home.

This year Simpson et al (1977) reported a randomised control study of hospital stay after the more serious operations of cholecystectomy and vagotomy, where these were uncomplicated. When the patient satisfied strict criteria of safety, e.g. sound wound, mobilising, eating small meals, having the use of their bowels, afebrile for 48 hours, it was found the average 'right' duration of stay was 7.6 days, a saving of 2 days on the controls. They point out that this is an average, and some patients will obviously need to stay longer for medical or domestic reasons.

A further practical way of improving surgical turnover has been suggested by Dudley (1977) who advocates increased patient mobility for straight forward operations so that hospitals with spare capacity help out others which are temporarily or permanently overloaded. If all these measures were applied, I am confident there would be a substantial benefit to the country's overall waiting list, which surely must be a source of shame to most of us in the National Health Service.

**CANCER OF THE BREAST**

In an attempt to diagnose this malignancy early, screening clinics have been set up without adequate control of their value. Often this has meant mammography routinely, but many authorities doubt its value and the National Cancer Institute thinks it may be dangerous (1977). Nor is there clear evidence that early diagnosis affects the outcome. Baum (1977) in reporting an increase in the age-adjusted death rate both in the UK and in the USA despite educational and treatment programmes, feels that prognosis depends on predetermined variations in growth rate, infiltrative power and host reaction. In the actual management of a patient with carcinoma, Kaae & Johansen (1967) showed in a randomised trial of 666 patients that simple mastectomy with radiotherapy carried a similar prognosis to the more mutilating radical operation. The latter operation actually caused more local and regional recurrences.

**ANGINA SURGERY**

The relief of this distressing symptom has been attempted by surgical means for 50 years, usually with enthusiastic reports of success. Dimond et al (1960) were the first to perform a random trial on internal mammary ligation, which was the fashionable operation of the time. They showed that a sham operation gave equal relief of angina, increased exercise tolerance and less trinitrin consumption. The popular operation in this decade is saphenous vein by pass, and this is now big business in the USA costing $1 billion in 1976. Whilst relief of angina can often be obtained, there is no evidence from the paper of Murphy et al (1977) that life expectancy is altered — the 3 year survival being 87% for medical patients allocated at random with the surgical patients with 88% survival. Improvement in mortality is more likely to accrue from stopping cigarette smoking.

### MEDICAL AUDIT

Our colleagues in obstetrics have shown us in recent years, from the Confidential Enquiries into Maternal Mortality, how valuable a retrospective study can be in pointing out deficiencies in management. Similarly, research on asthma has pointed to the potential dangers of abuse of adrenaline inhalers. Two months ago Rose et al (1977) studied 116 deaths following head injury who were admitted to a neurosurgical unit in Glasgow. They found avoidable factors which may have contributed to death in 54%, the most common being delay in the treatment of an intracranial haematoma. I think we shall see more of this type of study, which high-light deficiencies in treatment. They do not involve greater expenditure but direct our attention to the priorities. The rewards will be some lives saved, and some invalidism prevented.

Another form of medical audit which seems beneficial to the public is peer review of operations. In Saskatchewan the number of hysterectomies had increased by 72% between 1964 and 1971 though there had been only a 7% increase in the female population over 15. The College of Physicians & Surgeons set up an investigating committee in 1972 and compiled a list of justified indications for the operation. By 1974 there had been a one-third reduction in hysterectomies, mostly by a marked fall in unjustified operations (Dyck et al 1977). It seems to me most important that the profession puts its own house in order in this way, with the resulting benefit accruing to the state and the individual.

**Table 2**

**Comparison between 1954/55 and 1967/68 in terms of spells of sickness commencing and total days of incapacity, standardised with equivalent 1951 population. Selected causes where a trend was present.**

**Males only (percentage)**

| | Days | Spells |
|---|---|---|
| Rises 1954/55 to 1967/68 | | |
| Sprains and strains | +267 | +228 |
| Nervousness, debility and headache | +189 | +139 |
| Psychoneuroses and psychoses | +152 | + 68 |
| Displacement of intervertebral disc | +147 | +171 |
| All injuries and accidents | + 72 | +109 |
| Falls 1954/55 to 1967/68 | | |
| Anaemias | – 12 | + 2 |
| Asthma | – 24 | – 6 |
| Skin diseases | – 24 | – 30 |
| Appendicitis | – 32 | – 41 |
| Pleurisy | – 44 | – 36 |
| Respiratory tuberculosis | – 83 | – 74 |

*From Cochrane, 1971*

### SICK ABSENCES & CERTIFICATES

You may have the impression that this country suffers from a lot of damaging industrial disputes. This is correct, but the days lost by such strikes pale into insignificance compared with those lost through certified sickness. In 1968 the ratio was 1:70 and the days lost through sickness have been increasing. The vision of Aneurin Bevan that a free service would improve the nation's health has not materialised. As can be seen in Table 2 there has been a remarkable increase in the time taken off for sprains and strains and nervous debility in particular. What was once endured is now the cause of some weeks off work. One cannot escape suggesting that one reason for this may be the too ready issue of certificates by the profession for disabilities which are relatively minor. Another explanation may be that behind this medical labelling there are other disorders such as alcoholism, laziness, personal frictions at work or boredom with job. A further contributory factor is that it is too easy not to work and that there are insufficient financial rewards from working hard. In 1951 a man off work drew on average 36% of his working wage, whereas in 1968 and today he draws about 75% (Table 3).

Clearly all these factors should be corrected, but the one which doctors can do most about is issuing certificates, which are often too freely given and for

**Table 3**

**Net weekly income at work and out of work (married couple with two children under the age of 11)**

| | 1951 | 1968 |
|---|---|---|
| (a) At work (average earnings for adult male manual workers in manufacturing, etc., industries and including Family Allowance and less National Insurance and Tax) | £8.24 | £20.03 |
| (b) Out of work (Sickness or Unemployment Benefit not subject to tax and including Earnings Related Supplement where applicable and Family Allowance) | £2.97 | £14.92 |
| (c) As percentages of (a) | 36.0 | 74.5 |

*From Cochrane, 1971*

too long. At the same time by prompt advice and treatment whether in surgery or hospital the time required to be away from work for genuine illness can be lessened. In hospital we must fight what I call 'clinicosis' — the excessive attendance at clinics whether by unnecessary follow-up or cross referral. An ex-registrar of mine has shown that prolonged follow-up of pulmonary tuberculosis patients after a period of adequate treatment is not necessary, and I am sure that this applies to many diseases, including malignancy.

### CONCLUSIONS

1. I do not consider that we need more doctors in this country. We are now training about 4,000 per year at £30,000 each and no longer export them to any degree. There is a real danger of over-doctoring and wasteful procedures leading to more iatrogenic diseases.
2. I do not think any greater slice of government expenditure should be devoted to health. Avoidance of wasteful procedures and the more effective application of our present resources should allow this containment of costs.
3. The cost of the administrative tiers must be reduced in all branches of the national health service, including the medical and nursing side. Time wasting and excessively large committees must be reduced to allow professional people to get on with their work.
4. Strict cost-benefit analysis in pilot centres should be obligatory before the widespread adoption of new techniques such as screening, new apparatus such as computerised records, new units such as for strokes or alcoholics.
5. New drugs, new treatments or operations should be subject to randomised controlled trials before their universal adoption. We should encourage medical audits of our work.
6. Reduce excessive invalidism by persuading the government that there must always be a strong financial incentive to work. Attempt to control excessive demands on medical services.
7. Curb the excessive drug bill by increasing the consumer's contribution to the cost, by prescribing cheaper preparations in smaller amounts, and by critical studies of effectiveness.
8. Recognise the truly doctorial or teaching role of the profession:
(a) to our patients on healthy habits of living, exercise, eating, behaviour and self care — 'your life in your hands'.
(b) to the wider public on safety in the home, on hazards in industry, on the benefits of clean air and fluoride in water.
(c) to each other by continuing postgraduate education not only of what is new, but what is harmful or useless and should therefore be discarded.

Perhaps I could conclude by the story of the six greatest men in the world: To Moses everything important was in the heavens, to Solomon it was in the head, to Jesus it was in the heart, to Marx it was in the gut. Everyone knows what Freud thought was important, and to Einstein it was all relative.

I may have overstated my case but perhaps you may see some relative merit in these arguments.

*The Long Fox Memorial Lecture, delivered in the University of Bristol on 24th November 1977.*

### REFERENCES

BAUM, M. 1977. The curability of breast cancer. *In* Breast Cancer Management — early and late. ed. B. A. Stoll. p. 3—13. London: Heinemann Medical.

BEDDOE, J. 1904. The Long Fox Lecture: The first annual lecture arranged by the Committee of The Long Fox Memorial, 4th November 1904. *Bristol Med. Chi. J.*, **22**, 303—320.

BEESON, P. B. 1974. Some Good Features of the British National Health Service. *Arch. Intern. Med.*, **133**, 708—713.

BOARDMAN, K. P. and GRIFFITHS, J. C. 1977. Elective out-patient surgery in orthopaedics. *Health Trends*, **9**, 9—11.

BRANDA, R. F., AMSDEN, T. W. and JACOB, H. S. 1977. Randomized Study of Nandrolone Therapy for Anemias Due to Bone Marrow Failure. *Arch. Intern. Med.*, **137**, 65—69.

BROD, J. 1977. The Rational Basis of Diagnosis in Internal Medicine. *J. Roy. Coll. Phycns.*, **11**, 323—324.

COCHRANE, A. L. 1971. Effectiveness and Efficiency — Random Reflections on Health Services. *The Rock Carling Fellowship.* The Nuffield Provincial Hospitals Trust.

DARBY, S., BENNETT, M. A., CRUICKSHANK, J. C. and PENTECOST, B. L. 1972. Trial of Combined Intramuscular and Intravenous Lignocaine in Prophylaxis of Ventricular Tachyarrhythmias. *Lancet*, **1**, 817—819.

DIMOND, E. G., KITTLE, C. F. and CROCKETT, J. E. 1960. Comparison of Internal Mammary Artery Ligation and Sham Operation for Angina Pectoris. *Am. J. Cardiol.*, **5**, 483—486.

DRONFIELD, M. W., McILLMURRAY, M. B., FERGUSON, R., ATKINSON, M., and LANGMAN, M. J. S. 1977. A prospective, randomised study of endoscopy and radiology in acute upper-gastrointestinal-tract bleeding. *Lancet*, **I**, 1167—1169.

DORAN, F. S. A., WHITE, M. and DRURY, M. 1972. The scope and safety of short-stay surgery in the treatment of groin herniae and varicose veins. *Brit. J. Surg.*, **59**, 333—339.

DUDLEY, H. A. F. 1977. Loosening Patient Immobility. *Lancet*. **I**, 1251—1253.

DYCK, F. J., MURPHY, F. A., MURPHY, J. K., ROAD, D. A., BOYD, M. S., OSBORNE, E., DE VLIEGER, D., KORCHINSKI, B., RIPLEY, C., BROMLEY, A. T. and INNES, P. B. 1977. Effect of surveillance on the number of hysterectomies in the province of Saskatchewan. *N. Engl. J. Med.*, **296**, 1326—1328.

FEGAN, W. G. 1963. Continuous Compression Technique of Injecting Varicose Veins. *Lancet*, **II**, 109—113.

FOX, E. L. 1894. On the Medical Man and the State. *Br. Med. J.*, **2**, 237—242.

GREENBERG, D. S. 1977. X-Ray Mammography: Silent Treatment for a Troublesome Report. *New Engl. J. Med.*, **296**, 1015—1016.

GRINER, P. F. and LIPTZIN, B. 1971. Use of the Laboratory in a Teaching Hospital: Implications for Patient Care, Eucation, and Hospital Costs. *Ann. Intern. Med.*, **75**, 157—163.

HALBERSTAM, M. 1976. Teaching Hospitals Are Obsolete. *Am. Med. News*, **2**.

HAMILTON, D. E., BYWATERS, E. G. L. and PLEASE, N. W. 1959. A controlled trial of various forms of physiotherapy in arthritis. *Br. Med. J.*, **1**, 542—544.

HAYES, M. J., MORRIS, G. K. and HAMPTON, J. R. 1974. Comparison of Mobilization after Two and Nine Days in Uncomplicated Myocardial Infarction. *Br. Med. J.*, **2**, 10—13.

HOPKINS, A. and SCAMBLER, G. 1977. How Doctors Deal With Epilepsy. *Lancet*, **1**, 183—186.

ILLICH, I. 1975. *Medical Nemesis: the expropriation of health.* London: Calder & Boyars.

INMAN, W. H. 1977. Study of fatal bone marrow depression with special reference to phenylbutazone and oxyphenbutazone. *Br. Med. J.*, **1**, 1500.

JUHL, E., CHRISTENSEN, E. and TYGSTRUP, N. 1977. The epidemiology of the gastrointestinal randomized clinical trial. *N. Engl. J. Med.*, **296**, 20—22.

KAAE, S. and JOHANSSEN, H. 1968. Simple versus radical mastectomy in primary breast cancer. *In* Prognostic factors in breast cancer. Proceedings of First Tenovus Symposium, Cardiff, 12—14 April 1967. eds. Forrest, A. P. M. and Kunkler, P. B. Edinburgh: E. & S. Livingstone.

KERN, F. 1976. Gastroenterology — 1976: Good News and Bad News. *Gastroenterology*, **71**, 537—541.

LORD, P. H. 1969. A Day-case procedure for the cure of third-degree haemorrhoids. *Brit. J. Surg.*, **56**, 747—749.

M.R.C. Working Party Report. 1969. Assessment of Short-term Anticoagulant Administration after Cardiac Infarction. *Brit. Med. J.*, **1**, 335—342.

MAHLER, H. 1977. Tomorrow's Medicine and Tomorrow's Doctors. *WHO Chronicle*, **31**, 60—62.

MATHER, H. G., MORGAN, D. C., PEARSON, N. G., READ, K. L. Q., SHAW, D. B., STEED, G. R., THORNE, M. G., LAWRENCE, C. J. and RILEY, I. S. 1976. Myocardial infarction: a comparison between home and hospital care for patients. *Brit. Med. J.*, **1**, 925—929.

McGARRY, J. 1977. Minimal delay out-patient Termination of Pregnancy. *Health Trends*, **9**, 7—9.

McKEOUN, T. 1976. The Role of Medicine: dream, mirage and nemesis. *The Rock Carling Fellowship.* London: Nuffield Provincial Hospital Trust.

MELIA, R. J. W., FLOREY, C. du V., ALTMAN, D. G. and SWAN, A. V. 1977. Association between gas cooking and respiratory disease in children. *Brit. Med. J.*, **2**, 149—152.

MITTRA, B. 1965. Potassium, glucose and insulin in treatment of myocardial infarction. *Lancet*, **2**, 607—609.

MOGENSEN, L. 1970. Ventricular Tachyarrhythmias and Lignocaine Prophylaxis in Acute Myocardial Infarction. *Acta Med. Scand.*, Suppl. **513**, 1—80.

MURPHY, M. L., HULTGREN, H. N., DETRE, K., THOMSEN, J. and TAKARO, T. 1977. Treatment of Chronic Stable Angina. *New Eng. J. Med.*, **297**, 621—627.

PENTECOST, B. L., MAYNE, N. M. C. and LAMB, P. 1968. Controlled Trial of Intravenous Glucose, Potassium and Insulin in Acute Myocardial Infarction. *Lancet*, **1**, 946—948.

PERRY, C. B. 1944. The Social Aspects of Acute Rheumatism. *Bristol Med. Chir. J.*, **59**, 1—10.

ROSE, J., VALTONEN, S. and JENNETT, B. 1977. Avoidable factors contributing to death after head injury. *Brit. Med. J.*, **2**, 615—618.

RUCKLEY, C. V., MACLEAN, M., LUDGATE, C. M. and ESPLEY, A. J. 1973. Major Outpatient Surgery. *Lancet,* **2,** 1193–1196.

SIMPSON, J. E. P., COX, A. G., MEADE, T. W., BRENNAN, P. J. and LEE, J. A. 1977. 'Right' stay in hospital after surgery: randomized controlled trial. *Brit. Med. J.,* **1,** 1514–1516.

STASON, W. B. and WEINSTEIN, M. C. 1977. Allocation of Resources to Manage Hypertension. *New Engl. J. Med.,* **296,** 732–739.

State of the Bristol Royal Infirmary for 1870. Bristol: Charity Universal, 1871.

THOMSON, J. L. G. 1977. Cost-effectiveness of an EMI Brain Scanner. A review of a 2-year experience. *Health Trends,* **9,** 16–19.

TULLOCH, J. A. and GILCHRIST, A. R. 1950. Anticoagulants in treatment of coronary thrombosis. *Brit. Med. J.,* **2,** 965–971.

UK collaborative study on alpha-fetoprotein. 1977. *Lancet,* **1,** 1323–1332.

Veterans Administration Cooperative Study Group on Anti-hypertensive Agents. 1972. Effects of Treatment on Morbidity in Hypertension. III Influence of Age, Diastolic Pressure and Prior Cardiovascular Disease; Further Analysis of Side Effects. *Circulation 45,* 991–1004.

WATERS, W. E. 1970. Controlled Clinical Trial of Ergotamine Tartrate. *Brit. Med. J.,* **2,** 325–327.

WILCOX, R. G. and MITCHELL, J. R. A. 1977. Intravenous Urography in the Management of Acute Retention. *Lancet,* **1,** 1247–1249.

